# Supplementary material for: Complexome profiling of the Chlamydomonas psb28 mutant reveals TEF5 as an early PSII assembly factor
Source: Plant Cell. 2025 Mar 17;37(6):koaf055. doi: 10.1093/plcell/koaf055 (PMC12164590; doi:10.1093/plcell/koaf055)
Supplement: koaf055_Supplementary_Data [file koaf055_supplementary_data.zip › psb28_interactive.zip!/plots/intensity/qualityPlots/peptides/COX4_Cre13.g567600.t1.2.html]

### Cre13.g567600.t1.2

Trivial name: COX4  
SeqCoverage [median]: 39.40  
Mass: 19.03 kDa  
  
 

| Sequence | MissedCleavage | Mass [Da] | wtIntensity | muIntensity | Score |
| --- | --- | --- | --- | --- | --- |
| DGEPPRQFVENGEFYVLK | 1 | 2123.03 | 0.00 | 7381.56 | 90.65 |
| FGTEEDPIIVPSLEAER | 0 | 1900.94 | 894.93 | 0.00 | 93.96 |
| HVEYIK | 0 | 787.42 | 4985.33 | 4562.88 | 110.54 |
| HVEYIKK | 1 | 915.52 | 581.50 | 0.00 | 114.72 |
| KVGDVLEAIEGGADK | 1 | 1499.78 | 3240.82 | 6572.20 | 54.28 |
| KVGDVLEAIEGGADKAK | 2 | 1698.92 | 7901.29 | 38518.39 | 104.21 |
| PSALSAEPPR | 0 | 1023.53 | 470.51 | 1257.38 | 143.03 |
| PSALSAEPPRK | 1 | 1151.63 | 2840.52 | 971.67 | 99.28 |
| QFVENGEFYVLK | 0 | 1471.73 | 0.00 | 2838.81 | 98.05 |
| VGDVLEAIEGGADK | 0 | 1371.69 | 16151.12 | 33270.43 | 167.20 |
| VGDVLEAIEGGADKAK | 1 | 1570.82 | 45843.76 | 155872.52 | 131.72 |
|  |  |  | 82909.8 | 251245.9 |  |
